# *Mycobacterium tuberculosis* TlyA Protein Negatively Regulates T Helper (Th) 1 and Th17 Differentiation and Promotes Tuberculosis Pathogenesis*

**DOI:** 10.1074/jbc.M115.653600

**Published:** 2015-04-06

**Authors:** Md. Aejazur Rahman, Parveen Sobia, Ved Prakash Dwivedi, Aakansha Bhawsar, Dhiraj Kumar Singh, Pawan Sharma, Prashini Moodley, Luc Van Kaer, William R Bishai, Gobardhan Das

**Affiliations:** From the ‡ School of Laboratory Medicine and Medical Science, University of KwaZulu-Natal, Durban, 4001 South Africa,; the ¶ North Eastern Region Biotechnology Programme Management Cell, Defense Colony, New Delhi, India,; the ‖ Department of Pathology, Microbiology and Immunology, Vanderbilt University School of Medicine, Nashville, Tennessee 37232,; the ** Center for Tuberculosis Research, Department of Medicine, Division of Infectious Diseases, Johns Hopkins School of Medicine, Baltimore, Maryland 21231-1001, and; the § Special Centre for Molecular Medicine, Jawaharlal Nehru University, New Delhi 110067, India

**Keywords:** cytokine, Mycobacterium tuberculosis, T helper cells, vaccine, virulence factor

## Abstract

*Mycobacterium tuberculosis,* the causative agent of tuberculosis, is an ancient pathogen and a major cause of death worldwide. Although various virulence factors of *M. tuberculosis* have been identified, its pathogenesis remains incompletely understood. TlyA is a virulence factor in several bacterial infections and is evolutionarily conserved in many Gram-positive bacteria, but its function in *M. tuberculosis* pathogenesis has not been elucidated. Here, we report that TlyA significantly contributes to the pathogenesis of *M. tuberculosis.* We show that a TlyA mutant *M. tuberculosis* strain induces increased IL-12 and reduced IL-1β and IL-10 cytokine responses, which sharply contrasts with the immune responses induced by wild type *M. tuberculosis*. Furthermore, compared with wild type *M. tuberculosis,* TlyA-deficient *M. tuberculosis* bacteria are more susceptible to autophagy in macrophages. Consequently, animals infected with the TlyA mutant *M. tuberculosis* organisms exhibited increased host-protective immune responses, reduced bacillary load, and increased survival compared with animals infected with wild type *M. tuberculosis*. Thus, *M. tuberculosis* employs TlyA as a host evasion factor, thereby contributing to its virulence.

## Teaser

**Background:** TlyA is a virulence factor in several bacterial infections and is evolutionarily conserved in many Gram-positive bacteria, but its function in *Mycobacterium tuberculosis* pathogenesis has not been elucidated.

**Results:** TlyA mutant *M. tuberculosis* induces enhanced host-protective Th1 and Th17 responses.

## Introduction

*Mycobacterium tuberculosis*, the causative agent of tuberculosis (TB),^3^ is responsible for millions of deaths each year throughout the world (1). *M. tuberculosis* is the most successful Gram-positive intracellular bacterial pathogen and primarily infects human lungs through the aerosol route. *M. tuberculosis* is phagocytosed by resident macrophages in the lungs, where it evades hostile killing mechanisms, and later it forms granuloma-like structures with recruitment of immune cells around infected macrophages (2, 3). Despite intense research on *M. tuberculosis* pathogenesis, detailed molecular mechanisms of the role of distinct mycobacterial virulence factors remain incompletely understood. To understand its mechanism of pathogenesis, the functions of numerous *M. tuberculosis* gene products are being characterized in animal models (3–5). The antigenic components that are absent in the vaccine strain Bacillus Calmette Guérin (BCG) to elicit critical protective immune responses against TB have been an area of intense research. Early secreted antigenic target protein-6 (ESAT-6) is one of the most prominent antigens expressed by *M. tuberculosis* but not by BCG (6, 7). Thus, ESAT-6 is being extensively studied for its potential activity as a subunit vaccine (6, 7). In continued efforts to search for virulence factors of *M. tuberculosis*, many researchers have identified clusters of genes that may serve as potential targets for vaccine development (8–10). Among the many unexplored gene products of *M. tuberculosis*, TlyA (Rv1694) was recently identified as a possible virulence gene in *M. tuberculosis* (11). A TlyA homologue is present in many pathogenic bacteria, and the encoded factors exhibit virulence-promoting properties by functioning as a pore-forming hemolysin in *Serpulina hyodysenteriae* and *Helicobacter pylori,* and with adherence properties to host cells or tissues in many pathogens (11–13). Moreover, the *TlyA* gene is also present in several pathogenic mycobacterial species, including *M. tuberculosis* and *Mycobacterium leprae*. Although, *M. tuberculosis* and *M. leprae* evolved from a common ancestor, *M. leprae* possesses fewer genes (14). Genes conserved between the two species are hence considered important for pathogenicity and virulence. Recently, Rahman *et al.* (11) reported that TlyA (Rv1694) of *M. tuberculosis* possesses hemolytic activity by binding with and oligomerizing into host cell membranes.

Macrophages are critical innate immune cells, engulfing microbes into phagosomes that later fuse with lysosomes containing enzymes that degrade the invaded organisms. This process also makes antigens available for priming of T cell responses (15–20). However, *M. tuberculosis* has evolved mechanisms to evade phagosome maturation and to alter the levels of cytokine secretion to ensure its unhindered survival and replication within phagocytes (17, 18). Mycobacterial replication in hosts facilitates recruitment of macrophages, epithelioid cells, and lymphocytes that ultimately leads to formation of granulomas that contain the organisms (21, 22). Furthermore, an equilibrium develops between the protective immune response and growth of the harbored mycobacteria, causing persistent infection. A later perturbation in immune responses may result in uncontrolled growth of *M. tuberculosis*, leading to symptomatic disease. Adaptive immunity against *M. tuberculosis* infection predominantly consists of interferon (IFN)-γ–producing CD4^+^T lymphocytes that activate macrophages to restore phagolysosome activation and enhance autophagy (23–25). IFN-γ is an essential component of the immunological defense against intracellular infections (26). Both mice and humans with genetic defects in IFN-γ signaling are highly susceptible to mycobacterial diseases (27). It has been established that T helper 1 (Th1) cells producing IFN-γ play a central role in host immunity against *M. tuberculosis* infection, and this type of immune response is generated in the presence of interleukin (IL)-12 secretion by infected macrophages (27). IFN-γ-induced autophagosomes target *M. tuberculosis*-containing phagosomes for lysosomal destruction. Additionally, lysosomal degradation products are presented via MHC class II molecules to MHC class II-restricted CD4^+^ T cells, and these helper T cells orchestrate the specific immune response. However, *M. tuberculosis* promotes the differentiation of regulatory Th2 and Treg cells, and this is associated with inhibition of protective T cell responses in the host (28–30). *M. tuberculosis* has also developed several strategies to escape entry and destruction by phagolysosomes and macroautophagy and, hence, to be recognized by MHC class II-restricted CD4^+^ T cells.

Here, we report that TlyA assists *M. tuberculosis* survival in a mouse infection model by inhibiting Th1 cytokines (IL-12 and IFN-γ) as well as autophagy. Furthermore, deletion of the TlyA gene in wild type *M. tuberculosis* H37Rv impedes its pathogenicity in mice. Therefore, TlyA is a virulence factor for *M. tuberculosis* that deserves more in depth study and needs to be considered when designing TB vaccines and therapies.

## Experimental Procedures

### 

#### 

##### Ethics Statement

All animal experiments were performed according to the guidelines approved by the Institutional Animals Ethics Committee meeting held on 16 August 2010 and 28 January 2013 at International Centre for Genetic Engineering and Biotechnology (ICGEB) (approval numbers ICGEB/IAEC/IMM-22/2010 and ICGEB/AH/2013/01/IMM-34), New Delhi, India, and Department of Biotechnology guidelines, Government of India. All mice used for experiments were ethically sacrificed by asphyxiation with carbon dioxide according to institutional and Department of Biotechnology (Govt. of India) regulations.

##### Mice

BALB/c and C57BL/6 mice (6–8 weeks of age) were initially purchased from The Jackson Laboratory. ERK, TLR-2, and MyD88 knock-out mice on the B6 background were the kind gift, from Prof. Ruslan Medzhitov, Yale University. All animals were subsequently bred and maintained in the animal facility of the International Centre for Genetic Engineering and Biotechnology (ICGEB), New Delhi, India.

##### Bacteria

*M. tuberculosis* strain H37Rv was a kind gift from the Colorado State University repository. H37RvΔTlyA was a kind gift from Tanya Parish, University of Washington (13). H37Rv and H37RvΔTlyA were grown in 7H9 (Middlebrook, Difco) medium supplemented with 10% albumin, dextrose, and catalase (Difco) and with 0.05% Tween 80 and 0.5% glycerol, and cultures were grown to mid-log phase. Aliquots of the cultures in 20% glycerol were preserved at −80 °C, and these cryopreserved stocks were used for infections.

##### M. tuberculosis Infection of Mice and Estimation of Colony-forming Units (cfu)

Mice were infected with wild type mycobacterial strain H37Rv or mutant strain H37RvΔTlyA via the aerosol route using a Madison aerosol chamber (University of Wisconsin, Madison, WI) with its nebulizer pre-calibrated to deposit a total of ∼110 bacilli/lung/mouse to the lungs of each mouse as described previously (7, 22). Briefly, mycobacterial stocks recovered from a −80 °C freezer were quickly thawed and subjected to light ultrasonication to obtain a single cell suspension. Fifteen ml of the bacterial cell suspension (10 × 10^6^ cells/ml) was placed in the nebulizer of the pre-calibrated Madison aerosol chamber and administered to mice. One day after the aerosol exposure procedure, three randomly selected mice were sacrificed to determine the infectious dose in each experiment. To determine viable at various time points, lungs and spleens were harvested and homogenized in sterile PBS containing 0.05% Tween 80 and plated onto 7H11 Middlebrook (Difco) plates containing 10% oleic acid, albumin, dextrose, and catalase (Difco). Undiluted, 10-fold diluted, and 100-fold diluted lung and spleen homogenates were plated in duplicate on the above 7H11 plates and incubated at 37 °C for 15–21 days. Colonies were counted, and colony-forming units were estimated.

##### T Cell Proliferation Assay

Spleens were isolated from uninfected and H37Rv- and H37RvΔTlyA-infected mice. Spleens were macerated by frosted slides in 10% RPMI 1640 medium (Gibco, Invitrogen) and made into a single cell suspension. Red blood cells (RBCs) were lysed with RBC lysis buffer and incubated at room temperature for 3–5 min and washed with 10% RPMI 1460 medium. Cells were counted, and 2 × 10^5^ cells per well were seeded in 96-well plates and stimulated with different concentrations of *M. tuberculosis* complete soluble antigen. Cells were cultured for 48 h and then pulsed with tritiated thymidine (^3^H-labeled tritiated thymidine, 1.0 μCi per well; Amersham Biosciences). One day later, cells were harvested on filter mats using a semi-automated cell harvester (PerkinElmer Life Sciences). Thymidine incorporation was determined by using a plate β-counter (PerkinElmer Life Sciences).

##### Flow Cytometry, Surface and Intracellular Staining

Spleens were isolated from H37Rv- and H37RvΔTlyA-infected mice and macerated by frosted slides in 10% RPMI 1640 medium (Gibco, Invitrogen) and made into a single cell suspension. RBCs were lysed with RBC lysis buffer, incubated at room temperature for 2–3 min, and washed with 10% RPMI 1640 medium. The cells were counted, and 1 × 10^6^ cells were used for surface staining. Cells were harvested and washed twice with PBS and stained with fluorescent antibodies directed against surface markers. For intracellular staining, 1 × 10^6^ cells were cultured per well in 24-well plates (Corning, Co-star) and activated with 50 ng/ml phorbol 12-myristate 13-acetate (Sigma) and 750 ng/ml ionomycin (Sigma) overnight, and 10 mg/ml brefeldin A (eBiosciences) was added during the last 3 h of culture. After staining, cells were washed again with PBS, and cells were fixed with 100 μl of fixation buffer (eBiosciences) for 15 min, then resuspended in 200 μl of 1× permeabilization buffer (eBiosciences), and stained with fluorescently conjugated monoclonal antibodies as follows: anti-IL-4 (clone, 8D4-8), anti-IL-17 (clone, 17B7), and anti-IFN-γ (clone, XMG1.2), all from Pharmingen. Fluorescence intensity of fluorochrome-labeled cells was acquired and analyzed by flow cytometry (FACS Canto II, BD Biosciences). Data analysis was performed by Flow Jo (Tree Star).

##### Generation of Dendritic Cells

C57BL/6 mice were euthanized, and the femurs were isolated. Bone marrow was flushed out with RPMI 1640 medium using a 2.0-ml syringe (26-gauge). The cells were washed twice with PBS and then cultured in complete RPMI 1640 medium (Gibco) supplemented with GM-CSF (40 ng/ml) and IL-4 (10 ng/ml) on 24-well plates (1 million cells/ml). On the 3rd day, 75% of the medium was replaced with fresh DC culture medium. On day 5, the suspended cells were removed, and the loosely adherent cells were collected as immature DCs. Flow cytometric analysis by using anti-CD11c, -CD11b, -CD80, -CD86, -MHC class II, and -IgG2a (isotype control) antibodies suggested that 95% of the cells were conventional DCs.

##### Detection of Cytokines

Bone marrow cells were isolated from mice (BALB/c), differentiated into immature DCs as described above, and cultured in 24-well plates (1 million cells/well). Cells were infected with H37Rv or H37RvΔTlyA at a multiplicity of infection (m.o.i.) of 1:10. Supernatants from cells were collected at 24, 48, and 72 h for cytokine profiling. Cytokines in the culture supernatant of DCs were assayed by a Luminex microbead-based multiplexed assay using commercially available kits according to the manufacturer's protocol (BioPlex, Bio-Rad).

##### Histology

Lung tissues were stained with Acid Fast Bacilli stain and H&E dyes as described previously (7, 22). Data are presented here as the number of granulomas viewed under ×10 objective and ×10 ocular lens. Granulomas were counted from 25 different areas of the lungs of each mouse, and the bar graph represents the mean number of granulomas of 12 mice ± S.E. The results are representative of three independent experiments.

##### Preparation of Peritoneal Macrophages and Surface Labeling of Bacteria

C57BL/6 mice were injected intraperitoneally with 2 ml of 4% thioglycollate (Brewer modified, BBL, BD Biosciences). Five days later, peritoneal exudate cells were isolated from the peritoneal cavity by washing with ice-cold RPMI 1640 medium supplemented with 10% fetal bovine serum (FBS, Thermo Scientific HyClone). Cells were cultured overnight at 37 °C, 5% CO_2_, and washed with RPMI 1640 medium, 10% FBS to remove nonadherent cells. Adherent monolayer cells were used as peritoneal macrophages. Surface labeling of H37Rv and H37RvΔTlyA with FITC was performed according to standard methods (31), just prior to infection. The bacteria were harvested, washed twice with PBS, pH 7.4, and resuspended with 0.1 m sodium carbonate buffer, pH 9.5, containing FITC (1 mg/ml, Sigma); the mixture was incubated for 30 min at room temperature with gentle shaking. The bacteria were collected by centrifugation, washed three times, suspended in RPMI 1640 medium (without antibiotics), and passed through a 26-gauge needle. Peritoneal macrophages were plated in 12-well plates (∼1 × 10^6^ cells/well), and infected with FITC-labeled bacteria at an m.o.i. of 10:1. The purity of macrophages obtained from thioglycollate-elicited peritoneal exudates and infections with FITC-labeled H37Rv and H37RvΔTlyA in macrophages was examined by flow cytometry (FACS Canto II; BD Biosciences), and data were analyzed with FlowJo.

##### Cell Viability Assay

Peritoneal macrophages, uninfected or infected with *M. tuberculosis*-WT or *M. tuberculosis*-ΔTlyA, were cultured for 48 h. Cell viability was determined by flow cytometric analysis of cells stained with propidium iodide at a concentration of 10 μm.

##### Measurement of Autophagy

Peritoneal macrophages were plated in 12-well plates (1 × 10^6^ cells/well) and infected with FITC-labeled H37Rv or H37RvΔTlyA. Before harvesting, the medium was removed, replenished with medium containing 75 ng/ml of LysoTracker Red DND-99 (32), and incubated at 37 °C, 5% CO_2_ for 15 min. Finally, the control and treated cells were harvested with trypsin/EDTA and washed in 1 ml of cold PBS. For LC3II staining, we infected the peritoneal macrophages with H37Rv and H37RvΔTlyA, and 48 post-infection, we removed the cells and stained them with anti-LC3II antibodies. The stained cells were analyzed by flow cytometry on channel APC-A for LysoTracker and FITC-A for the LC3II staining using the FACS Canto II cytometer (BD Biosciences).

##### Confocal Microscopy

Peritoneal macrophages were plated in 12-well plates (∼1 × 10^6^ cells/well) on glass coverslips and infected with FITC-labeled bacteria at m.o.i. of 10:1. Bacteria were briefly sonicated before infection. After 4 h of infection, cells were washed twice with RPMI 1640 medium, 10% FBS and treated with 100 μg/ml gentamicin for 1 h at 37 °C to remove extracellular bacteria. After 1 h, medium was replaced with fresh RPMI 1640 medium, 10% FBS, and cells were cultured at 37 °C and 5% CO_2_. The medium was removed, and fresh medium containing 2 μm LysoTracker Red DND-99 was added and incubated for 2 h at 37 °C. Medium was removed from the wells, washed twice with PBS, and cells were fixed with 2% paraformaldehyde in PBS, pH 7.4, for 20–30 min. After fixation, cells were washed three times with PBS, and coverslips were transferred into fresh plates and stored at 4 °C until use. Coverslips were mounted in Prolong Gold antifade reagent (catalog no. P36934; Invitrogen) and sealed using adhesives. Confocal microscopy images were acquired on a Nikon A-1R confocal microscope with ×60 (for statistical data) and ×100 (for images) objectives and analyzed using NIS element software. For each sample, triplicate slides were prepared, and for each slide at least four different fields containing ∼10–15 cells were analyzed. Co-localization studies were performed after background correction using the NIS element software.

##### Western Blot Analysis

Western blotting was performed to detect the expression of TRAF-6. Whole cell lysates were prepared from peritoneal macrophages uninfected or infected (H37Rv or H37RvΔTlyA) by using lysis buffer (50 mm Tris-HCl, pH 8.0, 5 mm EDTA, 150 mm NaCl, 1% Nonidet P-40, 1 mm dithiothreitol, and 1 mm phenylmethylsulfonyl fluoride) along with phosphatase inhibitor mixture (78420; Thermo Scientific) and protease inhibitor mixture (78410; Thermo Scientific). Samples were electrophoresed on a 12% SDS-polyacrylamide gel and electroblotted onto polyvinylidene difluoride (PVDF) membranes. Blots were blocked for 2 h in 5% nonfat dried milk in TBST (1 m Tris, pH 8.0, 4 m NaCl, and 0.1% Tween 20). TRAF-6 protein was detected with anti-TRAF-6 (H-274 and Sc-7221) antibodies at a dilution of 1:500 for 2 h at room temperature. Bound antibody was detected with goat anti-rabbit (Sc-2004, Santa Cruz Biotechnology) antibody at a dilution of 1:1000 for 1 h at room temperature. Blots were stripped and re-probed for GAPDH as an equal loading control.

##### qPCR Analysis

Bone marrow-derived DC were isolated and infected with different bacterial strains (H37Rv and H37RvΔTlyA) and cultured for 48 h for RNA isolation. Total RNA, including miRNAs, was isolated by miRNeasy isolation kit (Qiagen, Germany) according to the manufacturer's instructions. cDNA was synthesized by the miRCURY LNA universal reverse transcriptase microRNA cDNA synthesis kit (EXIQON), and the reaction was set up according to the manufacturer's protocol. Real time quantitative RT-PCR analysis was performed using real-time thermal cycler (Bio-Rad) and miRCURY LNA universal reverse transcriptase microRNA PCR SYBR Green master mix (EXIQON, Vedbaek, Denmark) for miRNA amplification. Fluorescence data were collected at each amplification step. The relative expression level of miRNAs was normalized to that of internal control 5S rRNA by using 2-ΔΔ*Ct* cycle threshold method.

##### Statistical Analysis

All data were derived from at least three independent experiments. A value of *p* < 0.05 was accepted as an indication of statistical significance. For all statistical analyses, Student's *t* test was performed to compare two groups.

## Results

### 

#### 

##### TlyA Is a Virulence Determinant in M. tuberculosis H37Rv

**FIGURE 1.**
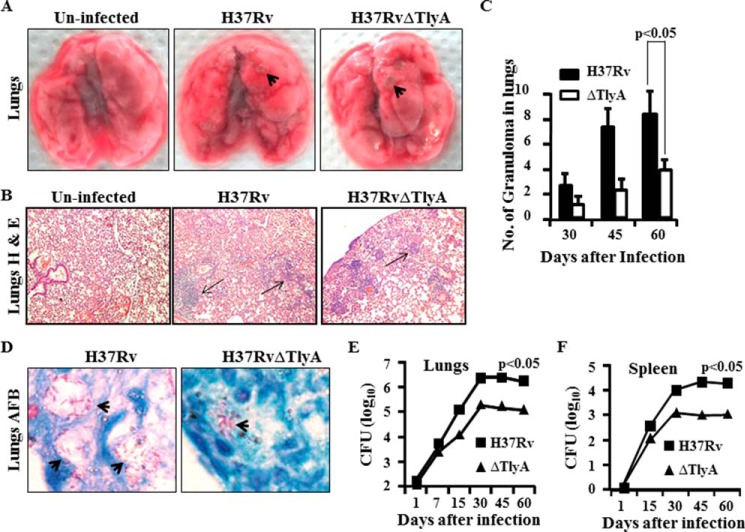
**General analysis of lungs and spleen of H37Rv- and H37Rv**Δ**TlyA-challenged BALB/c mice.** BALB/c mice were challenged with H37Rv or H37RvΔTlyA by the aerosol route, and lungs and spleen of the infected mice were harvested at different time points. *A,* gross pictures of lungs of uninfected, H37Rv-infected, and H37RvΔTlyA-infected mice. *B,* histology of the lung tissue sections at 45 days post-infection stained with hemotoxylin & eosin. *C,* numbers of granulomas in lungs at different time points after infection. *D,* acid fast staining of bacilli. *E,* colony-forming units from the lung homogenates of H37Rv- and H37RvΔTlyA-infected mice at different time points. *F,* colony-forming units from splenocytes of mice that were infected with H37Rv or the H37RvΔTlyA mutant. The results shown are representative of three independent experiments with five mice per group per time point.

Previously, it has been reported that TlyA is a virulence factor in many pathogenic bacteria. Consistent with its virulence properties, TlyA possesses hemolytic activity in several organisms and assists *H. pylori* to adhere to and colonize gastric epithelial cells (11).To investigate the role of TlyA in *M. tuberculosis* infection, we infected mice with *M. tuberculosis* H37Rv or a TlyA mutant of H37Rv (H37RvΔTlyA), using a low dose (∼110 cfu) infection model by the aerosol route. At different time points after infection, we harvested lungs and spleen for analysis of bacterial load and cytokine expression. We found that mice infected with H37RvΔTlyA developed lower numbers of granulomatic lesions than mice infected with H37Rv (Fig. 1*A*). These results were further strengthened by histological analysis, which showed lower numbers of granulomatic regions and bacilli in lungs of mice infected with H37RvΔTlyA, compared with H37Rv (Fig. 1*B*), as well as numbers of granulomas in the lungs (Fig. 1*C*) and the presence of acid fast bacilli in the lung sections (Fig. 1*D*). Interestingly, H37RvΔTlyA and H37Rv replicated to a similar extent during the first 2 weeks of infection; however, at later time points, growth of H37RvΔTlyA bacilli gradually slowed down and reached a plateau in both lungs and spleen (Fig. 1, *E* and *F*). This observation suggested that adaptive immune responses play an important role in diminished growth of H37RvΔTlyA in organs. Therefore, TlyA is a virulence determinant of *M. tuberculosis* H37Rv, which assists *M. tuberculosis* in host evasion by controlling adaptive immune responses.

##### H37RvΔTlyA Induces Enhanced Antigen-specific T Helper Cell Activation

**FIGURE 2.**
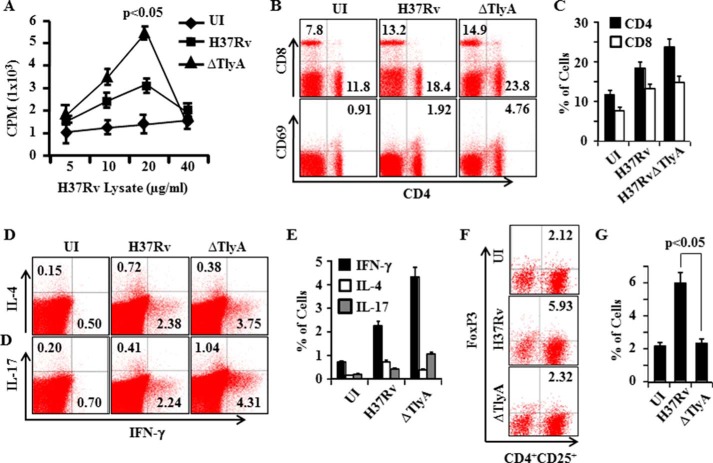
**T cell proliferation and FACS analyses of T cell subsets.** *A,* T cell proliferation from spleen of H37Rv- and H37RvΔTlyA-infected mice. *B* and *C,* FACS analysis shows the percentage of CD4^+^T cells, CD8^+^T cells, and activation marker CD69- and CD25-positive cells in *M. tuberculosis*-infected mice. *D* and *E,* T cells secreting IFN-γ, IL-4, or IL-17 among splenocytes of *M. tuberculosis*-infected mice. *F* and *G*, percentage of Treg cells (FoxP3^+^ CD4^+^CD25^+^cells) among splenocytes of *M. tuberculosis*-infected mice. The results shown are representative of three independent experiments with three mice per group per time point. *UI, uninfected.*

*M. tuberculosis* maintains an unhindered lifestyle within susceptible hosts by evading host protective immune responses. In addition to its innate immune evasion mechanisms within macrophages, *M. tuberculosis* is also successful in modulating adaptive immune responses. Above, we showed that H37RvΔTlyA mutants only exhibited enhanced clearance during the late phase of infection, suggesting that TlyA plays a role in inhibiting adaptive immune responses during disease progression. It is well accepted that Th1 and Th17 cells play a central role in host protection against *M. tuberculosis* infection. Therefore, we examined the status of adaptive immune components in animals infected by H37RvΔTlyA. As expected, we observed significantly higher T cell proliferative responses upon i*n vitro* challenge with complete soluble *M. tuberculosis* antigen in mice infected with H37RvΔTlyA, compared with H37Rv (Fig. 2*A*). This was also reflected by the prevalence of activated CD4^+^T cells and CD8^+^T cells in H37RvΔTlyA-infected mice, as deduced by the numbers of CD69-expressing cells. These results suggested that deletion of TlyA in H37Rv promotes activation and proliferation of antigen-specific CD4^+^T cells in infected animals (Fig. 2, *B* and *C*). It is now clear that Th1 and Th17 cells play host-protective roles, whereas Th2 and Treg cells potentiate disease progression. Therefore, we evaluated whether H37RvΔTlyA induced a biased T helper response. Indeed, we found that H37RvΔTlyA-infected animals produced dramatically higher numbers of IFN-γ- and IL-17-producing cells, whereas IL-4-producing cells were significantly reduced as compared with H37Rv-infected mice (Fig. 2, *D* and *E*). We also observed that H37RvΔTlyA induced significantly reduced Treg responses compared with H37Rv (Fig. 2, *F* and *G*).

##### H37RvΔTlyA Induces Both Th1- and Th17-mediated Immune Responses

**FIGURE 3.**
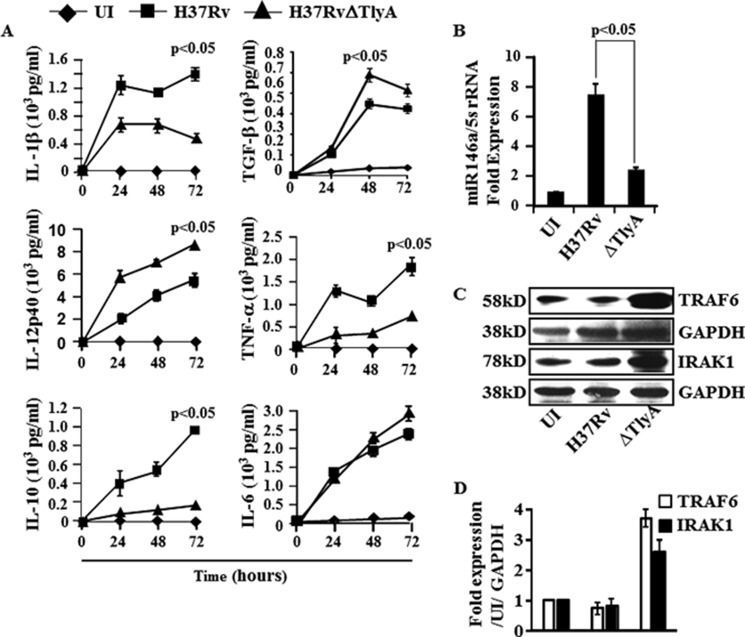
**Cytokine profiling of H37RvΔTlyA-infected dendritic cells.** *A,* DCs of mice were infected with H37Rv or H37RvΔTlyA, and cytokines from culture supernatant were assayed at different time points and compared with uninfected (*UI*) DCs. Luminex assay showing the cytokine concentration of IL-1β, IL-10, IL-12p40, IL-6, TGF-β, and TNF-α in the culture supernatants of H37Rv-infected (■) or H37RvΔTlyA-infected (▴) DCs compared with uninfected DCs (♦). The results shown are representative of at least three independent experiments with three replicates. *B,* miR146a expression in H37Rv- and H37RvΔTlyA-infected DCs after 48 h of infection. The results shown are representative of at least three independent experiments with three replicates. *C* and *D,* total cell lysates from uninfected or *M. tuberculosis* (H37Rv or H37RvΔTlyA)-infected macrophages were electrophoresed on 12% SDS-polyacrylamide gels and transferred onto nitrocellulose membranes. These membranes were probed with anti-TRAF-6 antibodies, then stripped and reprobed with anti-GAPDH as an equal loading control. Immunoblots are representative of three independent experiments.

From the above section, it is clear that deletion of the TlyA gene from H37Rv results in significantly higher Th1 and Th17 cytokine-producing cells, indicating that TlyA directly or indirectly regulates these host-protective immune responses. To provide insight into the mechanism whereby deletion of TlyA promotes Th1 and Th17 differentiation, we compared the cytokines induced by dendritic cells (characterized with CD11c, CD11b, CD80, CD86, and MHC class II markers) infected with H37Rv or H37RvΔTlyA. We found that H37Rv-infected DCs produced significantly higher amounts of IL-1β, IL-10, and TNF-α than DCs infected with H37RvΔTlyA (Fig. 3*A*). Additionally, we observed significantly higher amounts of IL-12p40, a key component of both IL-12p70 and IL-23, which are the growth factors for Th1 and Th17 cells, respectively, in the supernatant of DCs infected with H37RvΔTlyA, compared with DCs infected with H37Rv (Fig. 3*A*). We also noticed that both H37Rv and H37RvΔTlyA induced similar amounts of IL-6 and TGF-β (Fig. 3*A*), which orchestrate Th17 cell differentiation. These observations suggested that deletion of TlyA from H37Rv creates an environment that is conducive for the differentiation of both Th1 and Th17 cells. Therefore, we concluded that TlyA inhibits Th1 and Th17 cell differentiation, either directly or indirectly.

To investigate the molecular basis for the failure of H37Rv to induce high levels of IL-12 in DCs, we compared induction of microRNA-146a, a negative regulator of innate immune components in infected DCs (33). Interestingly, we found that H37Rv significantly up-regulated miR146a in DCs as compared with H37RvΔTlyA-infected or uninfected DCs (Fig. 3*B*). Consistent with these findings, the virulent strain H37Rv but not its H37RvΔTlyA variant inhibited the expression of TRAF-6, a target of miR146a, in DCs (Fig. 3, *C* and *D*). Collectively, these data suggested that H37RvΔTlyA promotes the differentiation of both Th1 and Th17 cell responses by modulating miR-146a and TRAF-6 expression.

##### H37RvΔTlyA Induces Autophagy in Macrophages

**FIGURE 4.**
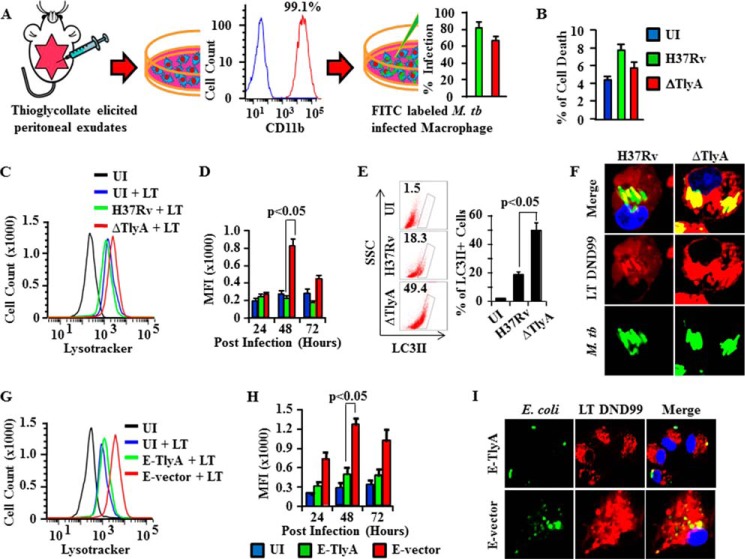
**TlyA induces autophagy in *M. tuberculosis* (*M. tb.*)-infected macrophages.** *A,* schematic model to show the purity of macrophages obtained from thioglycollate-elicited peritoneal exudates and the percentage of infected macrophages using FITC-labeled H37Rv and H37RvΔTlyA, as revealed by flow cytometry. *B,* cell death after 72 h was assessed by propidium iodide staining followed by flow cytometry. *C,* autophagy was assessed by measurement of LysoTracker Red DND-99 using flow cytometry. FITC-labeled H37Rv- and H37RvΔTlyA-infected macrophages were stained with DND-99 and analyzed on channel APC-A. Representative histograms are displayed. Data overlays were performed by FlowJo. The *y* axis shows cell counts, and the *x* axis represents fluorescence intensity in log scale and shifting of DND-99. *D,* mean fluorescence intensity (*MFI*) value of autophagy. *E,* flow cytometry data to show the percentage of LC3II expression. *F,* confocal microscopy of FITC-labeled H37Rv- and H37RvΔTlyA-infected macrophages. Infected macrophages were stained with DND-99 and viewed under a ×100 optical zoom. *G,* autophagy was assessed by measurement of LysoTracker Red DND-99 using flow cytometry. Macrophages infected with FITC-labeled TlyA-expressing *E. coli* (*E-TlyA*) or mock vector transformed *E. coli* (*E-vector*) were stained with DND-99 and analyzed on channel APC-A. Representative histograms are displayed. Data overlays were performed by FlowJo. The *y* axis shows cell counts, and the *x* axis represents fluorescence intensity in log scale and shifting of DND-99. *H,* mean fluorescence intensity value of autophagy. *I,* confocal microscopy of FITC-labeled E-TlyA- and E-vector-infected macrophages. Infected macrophages were stained with LysoTracker-DND-99 and viewed under a ×100 optical zoom. The results shown are representative of three independent experiments. *UI,* uninfected.

Previous reports have indicated that virulent strains of *M. tuberculosis* suppress autophagy to inhibit antigen presentation, which represents an astute mechanism of host evasion (24, 25). Furthermore, the cellular stress associated with an increased autophagic influx stimulates lysosomal proton pumping, thus establishing a correlation between autophagic activity and overall lysosomal acidity. This process promotes antigen presentation and, in turn, T cell priming. Therefore, we tested whether H37RvΔTlyA promotes autophagy. Fig. 4*A* illustrates our procedure to isolate viable macrophages from peritoneal exudates of mice and to infect these cells with H37Rv or H37RvΔTlyA. *M. tuberculosis*-infected macrophages were stained with propidium iodide to test for apoptotic cells by FACS analysis. We only found 6–8% apoptotic cells at 72 h post-infection in *M. tuberculosis*-infected macrophages, compared with 4% in uninfected cells (Fig. 4*B*). Autophagy in *M. tuberculosis*-infected macrophages was measured with LysoTracker DND-99 at different time points after infection. We found that H37RvΔTlyA-infected macrophages exhibited exaggerated autophagic activity compared with uninfected and H37Rv-infected macrophages (Fig. 4, *C* and *D*). Moreover, analysis of the kinetics of autophagy showed that H37RvΔTlyA-infected macrophages exhibited enhanced autophagy at 48 h post-infection compared with H37Rv-infected macrophages (Fig. 4*D*). To confirm these data, we also measured autophagy by LC3II expression (Fig. 4*E*). As expected, we observed higher LC3bII expression in H37RvΔTlyA-infected cells. These results were further strengthened by confocal microscopy, which showed that H37RvΔTlyA organisms co-localized with LysoTracker-stained acidic compartments in infected macrophages, although this co-localization was less profound for H37Rv (Fig. 4*F*). These results were further supported by studies with *M. tuberculosis-TlyA*-transformed *E. coli* bacteria, where we observed that TlyA-expressing *E. coli* showed reduced autophagy as compared with mock vector-transformed *E. coli* (Fig. 4, *G* and *H*). Confocal microscopy studies with TlyA-expressing *E. coli* failed to show co-localization with LysoTracker-stained acidic compartments in infected macrophages, whereas complete co-localization was observed in vector-transformed *E. coli* (Fig. 4*I*). These results indicate that TlyA of *M. tuberculosis* inhibits autophagy. This finding may explain why H37RvΔTlyA induces higher antigen-specific immune responses than the parental strain H37Rv.

##### TlyA Modulates the TLR-2-MyD88 Signaling Pathway in a p38-MAPK-dependent Manner

**FIGURE 5.**
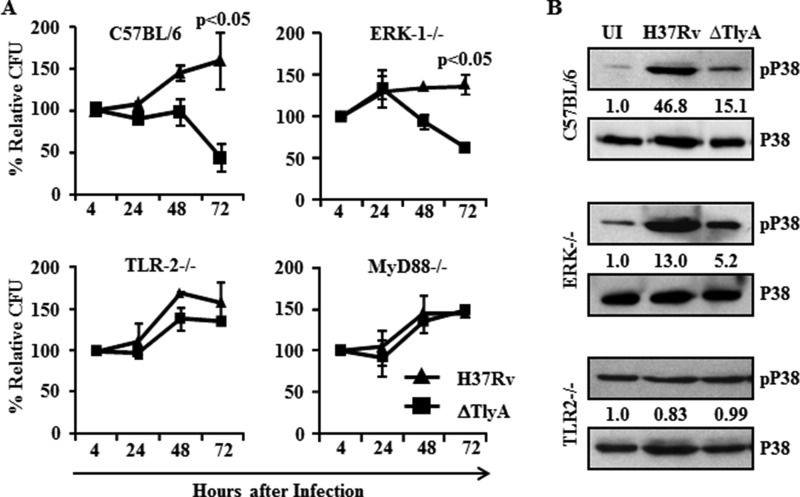
**TlyA activates p38-MAPK in the TLR2-MyD88 signaling pathway.** *A,* macrophages from wild type C57BL/6 and ERK^−/−^, TLR2^−/−^, and MyD88^−/−^ mice were infected with H37Rv or H37RvΔTlyA to determine the relative colony-forming units at different time points. The relative colony-forming units of H37RvΔTlyA was decreased in macrophages isolated from wild type and ERK^−/−^ mice at 72 h post-infection, compared with H37Rv. Moreover, colony-forming units of H37Rv and H37RvΔTlyA were similar to wild type in macrophages isolated from TLR2^−/−^ and MyD88^−/−^ mice. *B,* Western blot analysis for phospho-p38 in macrophages isolated from wild type C57BL/6 and ERK^−/−^ and TLR2^−/−^ mice infected with H37Rv or H37RvΔTlyA at 24 h post-infection. H37Rv-infected macrophages from wild type and ERK^−/−^ mice showed activation of p38-MAPK compared with uninfected (*UI*) and H37RvΔTlyA-infected macrophages. In contrast, p38-MAPK was equally activated in TLR2^−/−^ and wild type mice for uninfected and H37Rv-or H37RvΔTlyA-infected macrophages. The results shown are representative of three independent experiments with three replicates.

Our results clearly demonstrated that H37RvΔTlyA promotes Th1 and Th17 cytokine expression and autophagy. To determine the molecular mechanism of TlyA-mediated signaling in macrophages, we evaluated the viability of H37RvΔTlyA in macrophages isolated from wild type C57BL/6 and C57BL/6 mice selectively deficient in either ERK, TLR2, or MyD88. We isolated macrophages from wild type and ERK, TLR-2, and MyD88 knock-out mice, infected these cells with H37Rv or H37RvΔTlyA, and investigated bacterial loads at different time points post-infection. We found that, in both wild type and ERK knock-out macrophages, relative colony-forming units of H37RvΔTlyA were about 50% of the colony-forming unit levels of H37Rv-infected macrophages at 72 h post-infection (Fig. 5*A*). Furthermore, no differences were observed in relative colony-forming unit levels of H37RvΔTlyA-infected macrophages isolated from TLR-2^−/−^ and MyD88^−/−^ mice compared with H37Rv-infected macrophages (Fig. 5*A*). These results indicated that TlyA of H37Rv modulates the TLR-2-MyD88 signaling pathway in an ERK-independent manner.

It has been well established that many hemolysins and toxic proteins activate the p38-MAPK pathway through its phosphorylation. To examine p38 activation, we infected murine macrophages isolated from wild type, ERK^−/−^, TLR-2^−/−^, and MyD88^−/−^mice with H37Rv or H37RvΔTlyA. These infected macrophages were harvested after 48 h and assayed for p38 phosphorylation. We found that phospho-p38 levels in H37Rv-infected macrophages were higher as compared with H37RvΔTlyA-infected and uninfected macrophages (Fig. 5*B*). The phosphorylation pattern of p38 in ERK^−/−^ macrophages was similar to that in wild type macrophages, whereas no differences in phospho-p38 levels for H37Rv- and H37RvΔTlyA-infected and uninfected TLR-2^−/−^ macrophages were observed (Fig. 5*B*). These results suggest that TlyA modulates p38-MAPK activation downstream of the TLR-2-MyD88 pathway and that this is independent of the ERK pathway.

## Discussion

*M. tuberculosis* is the oldest known human pathogen and successfully co-evolved with vertebrate evolution. For its survival within macrophages, the natural host of *M. tuberculosis*, the organism inhibits phagolysosome fusion, neutralizes the lysosomal acidic environment, inhibits autophagy, and translocates to the cytosol (18, 24, 31, 32). Each of these measures assists the organism to evade innate immune responses. However, for its long term survival, *M. tuberculosis* also needs to modulate adaptive immune responses. Previous studies have shown that *M. tuberculosis* inhibits expression of MHC class II and co-stimulatory molecules, which can partially suppress T cell activation (34, 35). However, during its evolution *M. tuberculosis* evolved more sophisticated means to modulate host immune responses and has been shown to influence T helper cell responses (36–38). As Th1 and Th17 responses provide host protection against TB, *M. tuberculosis* inhibits these responses in susceptible hosts, which in turn enhance Th2 and Treg cell responses that assist disease progression (28, 29, 39). During its co-evolution with mammalian hosts, *M. tuberculosis* gradually acquired multiple means to evade various host-protective immune responses to avoid host elimination. Therefore, the virulence of *M. tuberculosis* is dependent on multiple host targets (40–42).

TlyA is a virulence determinant of many pathogenic bacteria such as *S. dysenteriae* and *H. pylori* (11–13). However, the role of TlyA in *M. tuberculosis* pathogenesis has not been previously investigated. Mycobacterial TlyA is present in virulent strains but absent in avirulent strains of *M. tuberculosis*. Interestingly, *M. tuberculosis-TlyA* shows high homology with the hemolysin/cytolysin TlyA of the swine pathogen *S. hyodysenteriae*, where TlyA was first characterized (12). The TlyA homologue in some bacteria exhibits hemolytic activity by forming pores, as confirmed in *S. hyodysenteria* (13), and TlyA in *H. pylori* functions as a hemolysin as well as an adherence factor for colonization of the gastric mucosa (12). The LsaA product of *Lawsonia intracellularis*, the TlyA homologue of this organism, lacks hemolytic activity but has been suggested to play a role in adherence and/or invasion (43). Moreover, TlyA is known as ribosomal RNA methyltransferase, which methylates 50S and 30S ribosomal RNA and makes *M. tuberculosis* susceptible to the peptide antibiotic capreomysin (12, 13, 44). Furthermore, TlyA has been shown to exhibit rRNA methyltransferase activity and to function as a hemolysin in *M. tuberculosis* (12, 13, 44). We observed that growth of H37RvΔTlyA in mice was similar to the parental strain during the initial phase of infection but that the bacteria were rapidly slowed down at later stages of infection. This observation suggests that adaptive immunity plays an important role in the inhibition of its growth. Furthermore, H37RvΔTlyA induced enhanced Th1 and Th17 responses in mice, suggesting that TlyA inhibits such responses during *M. tuberculosis* infection to subvert adaptive immunity. This modulation of adaptive immunity is mediated by cytokine regulation in infected cells. H37RvΔTlyA selectively alters expression of IL-12p40, suggesting that TlyA was evolutionarily acquired by mycobacteria to combat host immunity.

Earlier studies have indicated that *M. tuberculosis* infection in the murine macrophage cell line RAW264.7 inhibits phagosome maturation, whereas treatment of infected macrophages with IFN-γ induces autophagy (45). Treatment of macrophages with IFN-γ resulted in accumulation of the autophagy marker LC3II on endomembranes (46). ESAT-6 from *M. tuberculosis* directly inhibits IFN-γ production by human T cells. This inhibition was due to the induced phosphorylation and increased activity of p38 MAPK and independent of the activation of ERK or JNK (46). Furthermore, additional studies have suggested that *M. tuberculosis* induces p38 MAPK phosphorylation to inhibit phagosome maturation (47). After this activation, p38 MAPK negatively regulates autophagic maturation. However, inhibition of p38 by its inhibitor SB203580 was sufficient to activate autophagy maturation (48). It has been established that Gram-positive bacteria like *M. tuberculosis* that contain TLR2 ligands activate host cells via a signaling cascade involving TLR2, MyD88, IRAK4, TRAF6, and finally NF-κB (49). It has been shown that TLR2 signaling is required for phagolysosome maturation in macrophages in response to *M. tuberculosis* infection (50, 51). Our results further suggest that inhibition of IFN-γ in *M. tuberculosis* H37Rv-infected mice causes enhanced growth in organs as compared with H37RvΔTlyA-infected mice. Furthermore, H37Rv-infected murine macrophages showed reduced autophagy (Fig. 4, *C–E*), increased bacillar growth (Fig. 5*A*), and increased activation of p38 phosphorylation (Fig. 5*B*) compared with H37RvΔTlyA. Moreover, the increase in p38 activation induced by *M. tuberculosis-TlyA* was dependent on the TLR2-MyD88 signaling pathway. These findings also suggest that TlyA either directly binds with TLR2 or regulates expression of another TLR2 ligand. In this context, previous studies have shown that *M. tuberculosis* Esat-6 and lipoarabinomannan bind with TLR2 (52). Whether TlyA regulates the expression of these factors remains to be determined and is one of our future goals.

Previously, it has been shown that IL-1β, a pro-inflammatory cytokine, is necessary to impart protection against TB. Virulent *M. tuberculosis* strains cause more inflammation than avirulent strains. In our laboratory, we have further shown that H37Rv induces more IL-1β compared with its RD-1 deleted mutant (28). In another study, H37Rv infection in IL-1β knock-out mice showed 10-fold more colony-forming units in lungs at 7 weeks after infection compared with wild type mice (53). It has also been reported that, *H. pylori* induces expression of miR146a in gastric epithelial cells and proinflammatory cytokine secretion. Furthermore, overexpression of miR-146a reduced *H. pylori*-induced IL-8, TNF-α, and IL-1β levels (54).

In a recent study, Li *et al.* (55) showed that BCG-infected (10 m.o.i.) macrophages induce miR146a around 8-fold, but IL-1β expression was not analyzed. Furthermore, these investigators overexpressed miR146a in RAW264.7 cells around 100–200-fold (miR-146a mimics) followed by BCG infection (miR146a inhibitor), but there were no significant differences in the production of IL-1β (55). In our study we have shown 8- and 4-fold increased miRNA146a expression in H37Rv-infected DCs compared with uninfected and H37RvDTlyA-infected DCs, respectively, and this induction in miRNA146a expression was comparable with the study by Li *et al.* (55). Such an increased miRNA-146a expression was only seen at high IL-1β concentrations, suggesting that this negative feedback loop is only activated during severe inflammation and that this might be crucial in preventing potentially dangerous inflammation from spiraling out of control. However, examination of the mechanism showed that this was not caused by down-regulation of IRAK1 or TRAF6 but instead occurred at the translational level, through as yet unidentified mechanisms (56).

*M. tuberculosis-TlyA* is also a homologue of bacterial hemolysins and possesses pore forming hemolytic activities (11). Recently, we and others have shown that *M. tuberculosis* translocates from the phagolysosome to the cytosol of macrophages (32). As TlyA has pore forming activities and plays a role in the pathogenesis of *M. tuberculosis* and other organisms, it will be interesting to explore whether TlyA is involved in this process, which is one of our future goals.

In summary, we have demonstrated that the TlyA protein contributes to the pathogenesis of *M. tuberculosis* by inhibiting host-protective, adaptive immune responses. The increased clearance of H37RvΔTlyA, a deletion mutant of TlyA in the virulent *M. tuberculosis* strain H37Rv, is dependent on subversion of the adaptive immune response. Cytokine analysis revealed that this mutant induces enhanced host-protective Th1 and Th17 responses. Hence, TlyA warrants consideration for designing TB vaccines and therapies.

## Footnotes

3 The abbreviations used are:

TB: tuberculosis
m.o.i.: multiplicity of infection
BCG: Bacillus Calmette Guérin
DC: dendritic cell.
